# How does mode of travel affect risks posed to other road users? An analysis of English road fatality data, incorporating gender and road type

**DOI:** 10.1136/injuryprev-2019-043534

**Published:** 2020-04-06

**Authors:** Rachel Aldred, Rob Johnson, Christopher Jackson, James Woodcock

**Affiliations:** 1 Active Travel Academy; School of Architecture and Cities, University of Westminster, London, UK; 2 MRC Biostatistics Unit/Centre for Diet and Activity Research, University of Cambridge, Cambridge, UK; 3 MRC Biostatistics Unit, University of Cambridge, Cambridge, UK; 4 Centre for Diet and Activity Research, Unviersity of Cambridge, Cambridge, UK

**Keywords:** bicycle, motor vehicle occupant, pedestrian, cross sectional study, passenger, driver

## Abstract

**Background:**

Most analysis of road injuries examines the risk experienced by people using different modes of transport, for instance, pedestrian fatalities per-head or per-km. A small but growing field analyses the impact that the use of different transport modes has on other road users, for instance, injuries to others per-km driven.

**Methods:**

This paper moves the analysis of risk posed to others forward by comparing six different vehicular modes, separating road types (major vs minor roads in urban vs rural settings). The comparison of risk posed by men and women for all these modes is also novel.

**Results:**

Per-vehicle kilometre, buses and lorries pose much the highest risk to others, while cycles pose the lowest. Motorcycles pose a substantially higher per-km risk to others than cars. The fatality risk posed by cars or vans to ORUs per km is higher in rural areas. Risk posed is generally higher on major roads, although not in the case of lorries, suggesting a link to higher speeds. Men pose higher per-km risk to others than women for all modes except buses, as well as being over-represented among users of the most dangerous vehicles.

**Conclusions:**

Future research should examine more settings, adjust for spatial and temporal confounders, or examine how infrastructure or route characteristics affect risk posed to others. Although for most victims the other vehicle involved is a car, results suggest policy-makers should also seek to reduce disproportionate risks posed by the more dangerous vehicles, for instance, by discouraging motorcycling. Finally, given higher risk posed to others by men across five of six modes analysed, policy-makers should consider how to reduce persistent large gender imbalances in jobs involving driving.

## Introduction

Road safety analysis has traditionally measured per-mode injury risk per head of population. Some more recent research calculates these risks in relation to a travel exposure metric; e.g. per trip, km, or hour. This allows us to differentiate risk attached to the use of a given transport mode from the amount of usage.^1 2^

Most exposure-based research examines the risk that different road users have of being injured themselves.^3^ There is much less research examining the risk imposed on other road users (ORUs). This knowledge gap is problematic for policy and analysis, given ‘near universal agreement that society should take stronger measures to prevent its members from doing things that endanger others than […] things that endanger only themselves’ (Evans, p315).^4^ ^5–7^

Some initial literature examined risks posed to ORUs by specific subgroups, particularly older car drivers compared with younger drivers.^4 8 9^ Comparisons between modes have begun to appear in the policy literature. In the UK, Transport for London has started quantifying risk posed to ORUs by the use of different transport modes in London,^10^ drawing on earlier research comparing risk posed by vans and lorries.^11^

However, there remains little published comparative academic analysis. A rare example is Scholes *et al* 6 which compares risk posed by people driving (combining cars, taxis, private hire vehicles and minibuses) to that posed by people cycling, per-hour, including by age and gender. The authors find that drivers pose substantially higher per-hour risk to ORUs than cyclists.

Scholes *et al* 6 used travel survey-based data to differentiate by age and gender. England’s travel survey does not cover travel for commercial purposes (delivering goods or conveying vehicles or passengers), hence they could not include heavier vehicles, associated with relatively high risks.^10 12^ Our paper moves the debate on by including these and other vehicular modes in the comparison, alongside gender and road type.

## Methods

This study uses secondary datasets to estimate risk posed to ORUs by cycles, cars/taxis, vans, buses, lorries and motorcycles, segmenting by gender and road type, here defined as urban major, urban minor, rural major and rural minor (in UK road numbering, major are ‘A’ roads, and minor ‘B’, ‘C’ and ‘U’ (unclassified)). For this paper, we define risk as other-party fatalities per billion vehicle kilometres travelled. We are not seeking to apportion blame, but to identify the amount of fatalities associated with a given level of usage of different modes: conclusions from this can be drawn about the implications of changes in the use of transport modes, for instance, reduction or shifts from one mode to another. We report both rates computed from the raw numbers and expected rates obtained from a Poisson regression model.

This model was built using four key datasets from England for 2005–15: Stats19 police injury data, Road Traffic Statistics (RTS, for distance travelled by vehicle type, road classification, and urban/rural status), National Travel Survey (NTS, for trip distances by mode, gender and urban/rural residence) and Office for National Statistics (ONS) for population estimates by gender. We used Driver and Vehicle Licensing Agency (DVLA, GB, 2012 – earliest available year) to estimate gender mode split for vehicle types with substantial professional use.

The Stats19 database includes all road-traffic casualties recorded by police, in three files (casualty-level, vehicle-level and incident-level). Working at casualty level, we matched to the other two levels to identify vehicles involved and incident-level factors included in our analysis (road/area type). We included six vehicular modes: cycles, motorcycles, cars/taxis, vans, lorries and buses.

To calculate and compare risk posed to ORUs, we needed first to attribute each casualty to another vehicle involved. Hence, we have not included the 24% of fatalities where no other vehicle was involved, and excluded 3% with unknown vehicle type. Most remaining casualties (85% of all casualties and 75% of fatalities) only involve one other vehicle, and for these, each casualty was automatically associated with that other vehicle. For instance, for a cycle-car collision injuring both a car occupant and cyclist, the injured cyclist is assigned to the car, and the car occupant to the cycle.

For incidents involving multiple vehicles of the same size, each casualty was assigned to one other vehicle chosen at random. Where vehicles of different sizes were involved, we assigned each casualty to the largest other vehicle involved. As a sensitivity analysis, in online supplementary appendix 1 we provide headline results excluding collisions with more than one other vehicle involved (online supplementary figure 1). We excluded fatalities on motorways, which prohibit pedestrians, non-motorised and low-powered vehicles. This gave a dataset of 14 425 fatalities between 2005 and 2015 (69% of all fatalities), including 4509 pedestrians.

10.1136/injuryprev-2019-043534.supp1 Supplementary data

10.1136/injuryprev-2019-043534.supp2 Supplementary data

RTS provides data on total distance travelled by road type and vehicle mode in England, and we used this in all analyses. NTS provides individual-level trip distances for private-usage modes. These were used to estimate cycle, motorcycle and car/taxi distances by gender (for taxis, which make up a small proportion of the car/taxi total, we assumed the patterning of trip distance for each gender was like that of car drivers). For van driving, which unlike lorry or bus has significant private usage, we assumed that NTS data on the gender split of private van/lorry trip distance (5.69% by women) also reflected the gender split for professional van usage (this compared with 3.35% of specialist van driver licences being held by women, and 6.32% of van drivers being women according to the English Labour Force Survey (LFS) 2005-15). For lorry driving, 4% of heavy goods vehicle (HGV) full licence holders were female (DVLA figures), so we assumed 4% of lorry distance is driven by women. We assumed 8.25% of bus or coach distance is driven by women, this being the percentage of passenger carrying vehicle (PCV) full licences held by women (in LFS data, 8.54% of bus and coach drivers are women).

When estimating distance travelled by road type by gender for bus- and lorry-driving, we applied the male-to-female ratio of licence holders uniformly to the distance splits by road type in RTS. However, for modes with NTS data on trip distance by gender (car, cycle, motorcycle, van), we developed an independent set of heuristic, distance-based rules to partition each trip to different road types (see online supplementary appendix 1). We sequentially allocated (a fixed amount of) each trip’s distance first to minor urban (rural) roads, then urban (rural) major roads, then minor rural (urban) roads, then rural (urban) major roads and the remainder to motorways, for an urban (rural) resident. We estimated distance thresholds using a simple optimisation function to minimise divergence between RTS estimates and NTS estimates (scaled up to the population using ONS figures). Because of differences in trip distances, this led to gender differences in estimated usage of different road types for these modes.

When building our regression model, the predictors included all main effects and two- and three-way interactions between casualty severity, road type, mode and casualty mode for the overall rates, and interactions up to the third level between casualty severity, road type, mode, gender and casualty mode for the gender-segregated rates, excluding the three-way interaction between casualty severity, mode and casualty mode. These interactions were chosen to form the most complex models that could be fitted. We included the covariate “casualty severity” to strengthen information about the relative effects of the other predictors, leveraging the similarities between different injury severities, as specified in the terms of the model. However, in this paper we focus on fatalities, being more reliable than injuries, many of which are unreported.

The models assume that the number of injuries for each category comes from a Poisson distribution, where the logarithm of the mean is defined by a linear combination of the coefficients identifying each category. Additionally, the log distance travelled is included as an additive “offset” to the linear model for the log mean, to enable calculation of the expected number of injuries per-km distance travelled. The models return estimates and confidence intervals for the regression coefficients, hence for the expected number of injuries per distance travelled for each combination of predictors.

We checked the Poisson assumption by comparing the model with the corresponding negative binomial model. The negative binomial model was only computationally feasible when mode/casualty and mode/road type interactions were excluded. The resulting Akaike information criterion (AIC) is slightly greater than for the Poisson model (5059 vs 4812), suggesting that the terms that are excluded are more beneficial in terms of model fit than the additional flexibility in the distribution around observations. See online supplementary appendix 1 for further details of our Poisson model.

## Results

### Road user fatalities by road-user type and road type

Table 1 presents the number of fatalities resulting from collisions with different vehicle types, in absolute numbers and as a proportion of all fatalities on the road type. Cars or taxis are associated with two-thirds of fatalities, followed by lorries at 16.5%, vans at 9%, buses at 5.3%, motorcycles at 2.3% and cycles at 0.4%. The proportions vary by road type, with lorries associated with more fatal collisions on rural major roads relative to other road types (23.6% of fatalities on such roads), and buses with relatively more fatalities on urban major roads (9.3% of fatalities) and urban minor roads (7.8%).

**Table 1:** Total ORU fatalities by road-user type as a percentage of all fatalities on the road type

	Rural major	Urban major	Rural minor	Urban minor	Total
Car/taxi	3649 (62.9%)	1989 (63.5%)	1968 (73.2%)	1995 (71.2%)	9601 (66.6%)
Van	524 (9%)	238 (7.6%)	283 (10.5%)	261 (9.3%)	1306 (9.1%)
Lorry	1366 (23.5%)	492 (15.7%)	277 (10.3%)	243 (8.7%)	2378 (16.5%)
Motorcycle	95 (1.6%)	108 (3.4%)	62 (2.3%)	65 (2.3%)	330 (2.3%)
Bus	162 (2.8%)	290 (9.3%)	87 (3.2%)	219 (7.8%)	758 (5.3%)
Cycle	7 (0.1%)	14 (0.4%)	13 (0.5%)	18 (0.6%)	52 (0.4%)
Total	5803	3131	2690	2801	14 425

### Distance travelled by road-user type and road type

Table 2 presents distances travelled by mode. While cars dominate across all road types, other patterns differ. Lorries are 6.8% of traffic on rural major roads, and cycles just 0.1%, while on urban minor roads, lorries are 1.2% of distance, less than cycles (2.8%).

**Table 2:** Distance travelled (bn km) by mode and by road type, as a percentage of all distance travelled for each road type

	Rural major	Urban major	Rural minor	Urban minor	Total
Car/taxi	988.7 (78.2%)	639.7 (80.8%)	494.4 (78.4%)	832.6 (79.8%)	2955.5 (79.2%)
Van	170.3 (13.5%)	97.5 (12.3%)	99.1 (15.7%)	137.2 (13.1%)	504.2 (13.5%)
Lorry	86.2 (6.8%)	26.3 (3.3%)	14 (2.2%)	12.8 (1.2%)	139.3 (3.7%)
Motorcycle	10.3 (0.8%)	9.4 (1.2%)	7.6 (1.2%)	15.9 (1.5%)	43.2 (1.2%)
Bus	7.1 (0.6%)	11.5 (1.5%)	4.7 (0.7%)	16.2 (1.6%)	39.5 (1.1%)
Cycle	1.5 (0.1%)	7 (0.9%)	10.4 (1.7%)	29 (2.8%)	47.9 (1.3%)
Total	1264.1	791.4	630.3	1043.7	3729.6

### Risk per distance travelled by road-user type and road type: raw data

Table 3 presents risk by road-user type and by road type, defined as the number of fatalities associated with that vehicle and road type, divided by distance travelled.

**Table 3:** Estimated total ORU deaths per bn km travelled, by road-user type, by road type

	Rural major	Urban major	Rural minor	Urban minor	Total
Car/taxi	3.69	3.11	3.98	2.40	3.25
Van	3.08	2.44	2.86	1.90	2.59
Lorry	15.85	18.72	19.72	19.01	17.07
Motorcycle	9.21	11.46	8.12	4.10	7.63
Bus	22.78	25.23	18.59	13.49	19.18
Cycle	4.71	2.01	1.25	0.62	1.09

Across all road types, buses are associated with an average of 19.2 ORU deaths per bn vehicle km, followed closely by lorries (17.1). Despite their small size, motorcycles are associated with 7.6 deaths to ORUs per bn km. As motorcycling has a low mode share, this equates to 330 or 2.3% of ORU deaths. For cars/taxis (associated with most of the road fatality burden overall) there are 3.3 ORU deaths per bn km, while vans see 2.6 ORU deaths per bn km. Finally, cycles are associated with 1.1 ORU deaths per bn km travelled.

On different road types, distinct patterns emerge. Lorry driving poses similarly high risks on any road type, but there are three times more ORU fatalities per motorcycle km on urban major roads than on urban minor roads. Buses show a similar but smaller disparity, with each kilometre driven on major roads resulting in twice as many fatalities as on urban minor roads. For cycles, ORU fatalities per km ridden on rural major roads is much higher than on other road types. For cars, vans, and cycles, risks posed are generally higher on rural than on urban roads.

Finally, table 4 presents results for all road types for fatal, serious and slight injuries. Most road injuries are not reported to police in England, so unlike for fatalities, these figures do not reliably represent the full burden to ORUs of either type of injury (also, during this period police definitions of ‘serious’ injuries were more subjective than is now the case).

**Table 4:** Estimated reported ORU deaths, serious and slight injuries per bn km travelled, by road-user type

	Fatal	Serious	Slight
Car/taxi	3.25	49.68	436.49
Van	2.59	25.66	217.89
Lorry	17.07	62.46	427.17
Motorcycle	7.63	100.10	613.83
Bus	19.18	144.72	973.84
Cycle	1.09	32.79	182.92

### Risk per distance travelled by road-user type and road type: model-based estimates

**Figure 1:**
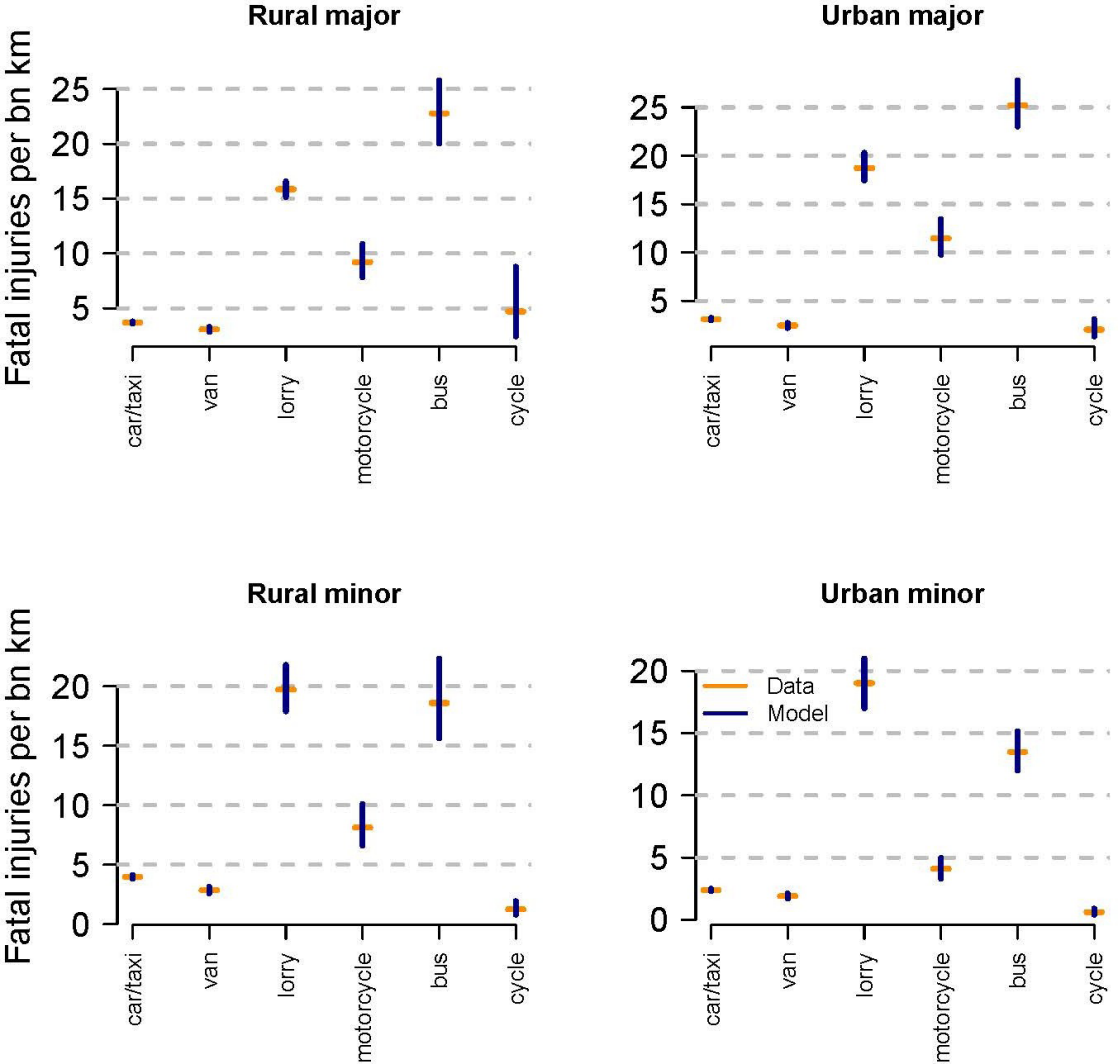
Relative risks to ORUs by mode, by road type (estimated risk from unsmoothed data, with 90% confidence intervals from the regression model).

Figure 1 presents the 90% CI for the modelled mean risks, comparing this with the unsmoothed rates presented above.

For cycles on rural major roads, low distance travelled and resultant ORU injuries means substantial uncertainty about risk rates. The mean modelled rate is 5.0 ORU deaths per billion km travelled, compared with 4.7 for the raw rate but with a 90% CI of 2.6 to 8.8. Of seven cyclist ORU fatalities on rural major roads, five were drivers or motorcyclists. We suspect this is linked to the relatively high speeds on these types of roads (of the seven roads, only one had a speed limit of 30mph, with the others being 50, 60, 60, 60, 60 and 70mph) and resultant higher risk for a driver in collision with a cycle.

### Gender

In table 5 we present risk calculations comparing males and females for each mode, providing both 90% confidence intervals for our main point estimate, and some simple sensitivity analysis for modes without NTS data. For cars and vans, the risk posed per km driven by men is double that posed by women, while for cycles the gap is slightly larger (the confidence intervals just barely overlap, but the chance of them being identical is low). For lorries, the risk posed by men is around four times higher, while for motorcycles, it is more than ten times higher. For buses, the risk posed by men is somewhat higher, but confidence intervals overlap.

**Table 5:** ORU fatalities by gender, with sensitivity analysis around distance assumptions

Mode	ORU fatalities per bn km			
	All road users	Men (90% confidence intervals by gender)	Women (90% confidence intervals by gender)	Women (sensitivity analysis for modes without NTS data)
Car/taxi	3.25	3.93 (3.85 to 4.00)	2.01 (1.94 to 2.07)	–
Van	2.59	2.62 (2.50 to 2.74)	1.32 (1.00 to 1.71)	–
Lorry	17.07	17.25 (16.7 to 17.9)	4.64 (3.23 to 6.33)	6.67
Motorcycle	7.63	8.18 (7.46 to 8.94)	0.68 (0.07 to 2.26)	–
Bus	19.18	19.45 (18.2 to 20.66)	14.35 (11.06 to 18.33)	20.64
Cycle	1.09	1.24 (0.96 to 1.56)	0.48 (0.18 to 0.97)	–

NTS data showed that for private van/lorry use, the gender gap in distance driven was more extreme than for any past-week use. Our estimate of van distance driven by women was 69.5% of what it would be had we assumed a distance ratio based on any past-week usage. This was used in the sensitivity analysis in table 5, that is, assuming women possessing an HGV or PCV licence drive 69.5% of the distance driven by their male counterparts. While not a like-for-like comparison this demonstrates the impact of plausible error. The overall picture remains similar, with substantially elevated risk posed by male lorry drivers, but bus driver risks more gender-equal (note that were 99% of lorry travel distance completed by male lorry drivers, the risk posed by each group would be equal).

**Figure 2:**
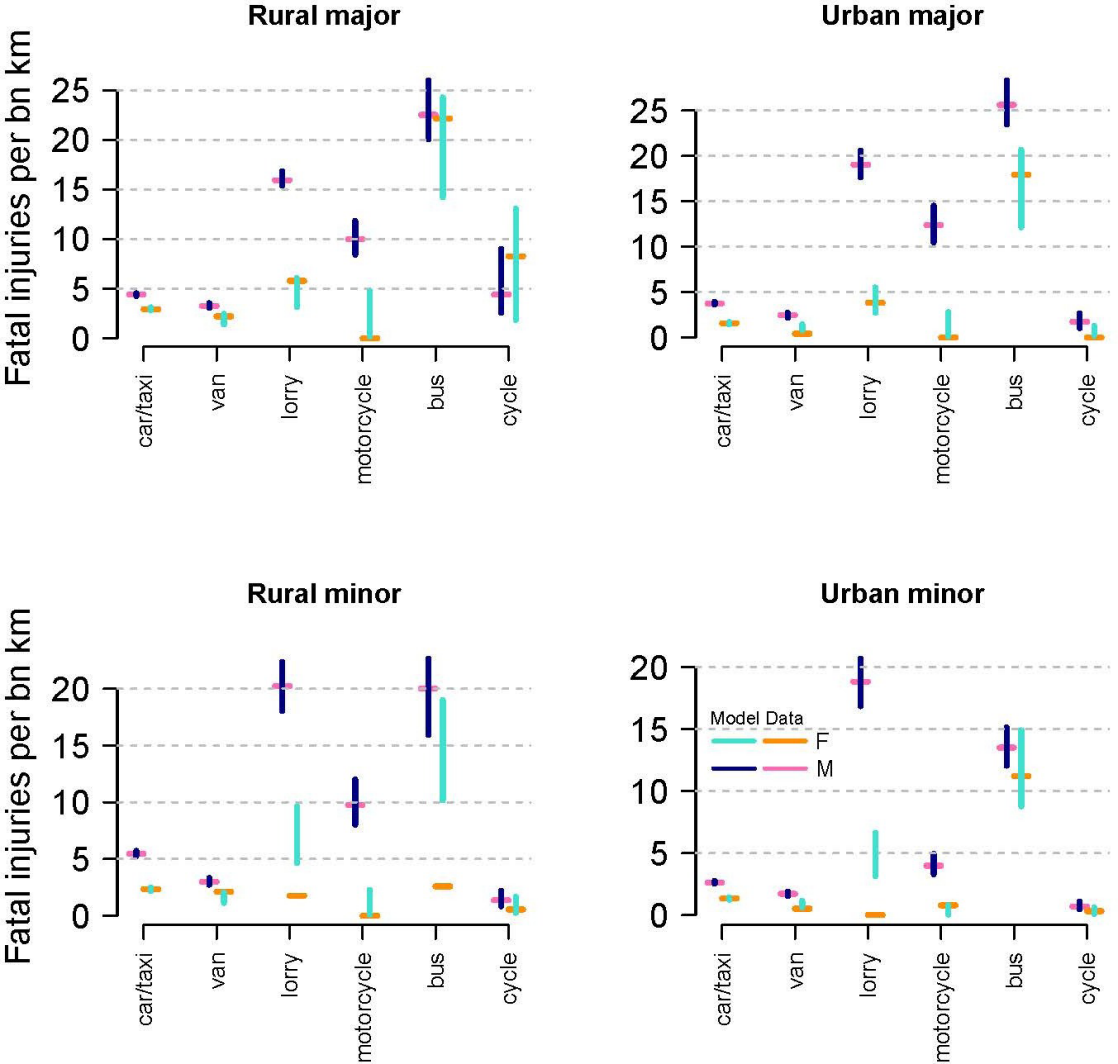
Gender breakdown of risk by mode and road type (estimated risk from unsmoothed data, with 90% confidence intervals from the regression model).

Figure 2 below illustrates modelled risk posed to ORUs by gender and road type with confidence intervals.

The risk posed by female motorcyclists, despite high uncertainty on each road type, never overlaps with the risk posed by males. For car/taxi, van and lorry driving, the confidence intervals do not overlap with male drivers posing more risk than females on all road types.

## Discussion

### Key findings

We found substantially higher ORU fatality risk associated with buses and lorries, compared with cars or vans. The risk posed by motorcycles lies in-between large vehicles and cars/vans. Cycles generate the lowest risk per km travelled. Major roads generate higher ORU fatality risk for most modes, except lorries. Male drivers/riders are associated with 2–4 times higher risk per km than female drivers/riders with two exceptions: buses with more similar risks, and motorcycles where men pose a much higher risk.

### Strengths and limitations

The article tackles an under-researched aspect of injury research and answers new questions. Notably, despite much analysis of risk experienced by motorcyclists^13^ previous academic analysis has not examined the risk motorcycling poses to ORUs.

Our use of traffic counts implies using a per km rather than per hour approach (giving a different perspective to Scholes *et al* 6), which enables us to provide more accurate disaggregation by road type. This still does not account for all spatial or any temporal correlations in the use of modes. Representing these multiple correlations is challenging given available data but could be a topic of future research.

Our confidence intervals only account for uncertainty arising from limited numbers of injuries per category. They exclude uncertainties about distance travelled, which would not be easy to calculate (RTS data provided by DfT remain estimates and breakdowns at Road Type, Vehicle and Region (England) level are not National Statistics). Traffic estimates for cycles are the least certain, as count locations have traditionally been established to monitor motorised movements. There may be smoothing errors inherent to the modelling process for the gender breakdowns, and these are particularly uncertain for work-related modes.

### Interpretations of our findings

#### By mode

Our findings confirm the analysis in Scholes *et al* 6 that a mode shift from driving to cycling would reduce risk posed to ORUs. A shift from motorcycling to cycling would have even greater injury benefits per driver/rider, especially as motorcyclists themselves experience^13^, as well as pose, high injury risk. More broadly, rather than only seeing motorcyclists as vulnerable, our analysis supports recognising the danger this activity poses to others: on urban roads, primarily pedestrians (135 of 173 motorcycle-related fatalities on urban roads).

The risk posed per km by buses and lorries are similar, despite the higher visibility and lower cabs on buses. This may be linked to inherent interactions with pedestrians at bus stops and along heavily walked routes, counteracting a safer vehicle design. Considered per person km, the risks posed to ORUs by buses and cars become comparable (given average bus occupancies of 12.5, and average car occupancies of 1.6: https://www.gov.uk/government/collections/bus-statistics). Despite many reasons why bus use is preferable to car use, it remains important to reduce the injury burden, especially as implementing changes to bus fleets or driving practices should be easier than for private cars.

Vans cause many fewer ORU deaths than lorries, per km travelled, with the gap particularly large on urban roads. As with buses vs cars, considering carriage the picture would change. A van can carry up to two tonnes (although many are used for servicing, not freight), but a large articulated lorry as much as 29 tonnes. These issues need to be taken into account when considering implications of the findings.

Vans appear slightly safer than cars per km driven. Given that many van drivers are experienced professional drivers, however, one might expect their risks to be lower still. Perhaps ‘gig economy’ working conditions mitigate against this by creating incentives to drive less safely.^14^ As each van driver will pose a high risk over a given time period (due to high mileage), interventions aimed at this group are worth considering.

#### By gender

For five of the six vehicular modes, Scholes *et al*’s finding for car use and cycling^6^ holds true: men pose more risk than women. Much literature has linked men’s relatively high crash involvement to gender differences in risk-taking,^15^ although the nature of this relationship is still debated.^16^ This paper highlights the persistence of the gap across most modes studied, alongside the preponderance of men (90%+) using the most dangerous modes, including in transport and related occupations.

For bus drivers, point estimates are closer with overlapping confidence intervals, although the point estimate remains higher for men. Perhaps characteristics of the vehicle and training and monitoring requirements may neutralise gender differences in skill or behaviour. However, substantial gaps exist for other modes, including vans and even lorries.

Motorcyclists have a very large gender risk gap, although uncertainty about risk posed by female motorcyclists is high given their low numbers. Part of this might be linked to gender differences in motorcycle type. This is suggested by a cross-tabulation of all motorcycles involved in collisions between 2005–15, from Stats19 injury data, as casualties or collision partners: 15% of those riding motorcycles under 50cc (basically mopeds) were women, but only 4% of those riding motorcycles over 500cc. Further research could focus on different types of motorcycle, to untangle the extent to which gender differences and/or vehicle size interact to produce the high risks found.

#### By road type

Analysis incorporating road type is novel and of interest given different speeds, speed limits, vehicular volumes and mixes and pedestrian volumes, all of which likely contribute to our results. England’s national speed limit is 60mph for single and 70mph for dual carriageway roads, for most vehicles. Most rural roads, minor or major, thus have speed limits of 60–70 mph. In urban areas, 30mph is the default limit, although some urban major roads have higher speed limits, and there are increasingly 20mph limits in residential streets or neighbourhoods.

The fatality risk to ORUs per km is generally higher on major than minor roads, although not for lorries. Reasons for this are likely to be complex. Higher traffic and pedestrian density is likely on major roads, hence more potential casualties, and this may vary by mode, by uncontrolled temporal and spatial factors. For instance, the proportion of cycling at peak times may vary by road type, if commuters disproportionately use major roads, and usage of different road types may be linked to behavioural or vehicular differences.

Speed could be an important factor, given that speeds are typically higher on major roads. This interpretation is supported by the relatively invariant fatality risk posed by lorries, which kill at low speeds, on the different road types; and by the generally higher risks posed by other vehicles on rural roads, which have typically higher speed limits. While more detailed research would help unpick the factors affecting risk by road type, these results provide some support for reducing speeds on major roads and in rural areas.

### Future research and policy implications

Further research could cover diverse country contexts with different casualty rates and mode shares, like Castro *et al*’s report on casualty risks experienced.^17^ Spatial data could be used to identify the types of road environments that heighten or mitigate risks posed to ORUs, drawing on similar work for risks experienced.^18^ Injury modelling studies can use these methods to incorporate impacts of mode shift in a sophisticated manner.^19^

There is a need to study further risks associated with the most dangerous vehicles and incorporate this and the results in policy. In road safety policy and analysis motorcyclists are traditionally seen as vulnerable road users (eg, https://www.highwaycodeuk.co.uk/road-users-requiring-extra-care.html) because of the high numbers of injured motorcyclists relative to mode share. This research by contrast highlights the high risk that motorcycle use poses to ORUs. Future research could further analyse the causes of this, by disaggregating different types of motorcycle: this could also help identify the primary cause of the much higher risk posed by male motorcyclists.

More research is needed studying the high risk posed by lorries and buses, as well as better data. Operators often have detailed data on fleet activity, including incidents and collisions (sometimes near misses). However, given the magnitude of these risks, arguably there is a public interest obligation on operators to make data available, and on public bodies letting contracts or franchises to incorporate such a duty.

Finally, we suggest that authorities should consider measures to reduce motorcycling, especially among men, and to deter motorcycling where it may otherwise be an unintended consequence of policy (for instance, of road pricing). More broadly, we suggest policy-makers consider policies to increase gender balance in occupations that substantially involve driving, given the greater likelihood that ORUs will be killed if men rather than women are driving or riding. Currently there is major gender imbalance in such occupations, and reduced risk to others could be a co-benefit from increasing gender equity.

**What is already known on the subject**

- Risk posed to other road users varies by mode and by demographic factors.
- Specifically, motor vehicle drivers pose higher risk than people cycling.
- Male car drivers and cyclists pose higher risk to others than their female counterparts.

**What this study adds**

- Identifies risk posed by use of a range of vehicles including lorries and motorcycles.
- Incorporates impact of road type to more accurately compare risk across modes.
- Men pose more risk than women and are more likely to use more dangerous modes.
