# Supplementary material for: How does mode of travel affect risks posed to other road users? An analysis of English road fatality data, incorporating gender and road type
Source: Inj Prev. 2020 Apr 6;27(1):71–6. doi: 10.1136/injuryprev-2019-043534 (PMC7848050; doi:10.1136/injuryprev-2019-043534)
Supplement: Supplementary data [file injuryprev-2019-043534supp001.pdf]

Appendix

1. Poisson model details & formula

Here we present the model for road-collision fatalities and injuries to ORUs as a function of the specifics of the crash and this distance travelled by the road user. This is a standard Poisson log-linear regression model for contingency tables,

$$I_{m,r,c,s} \sim Pois(\lambda_{m,r,c,s});$$
$$\lambda_{m,r,c,s} = D_{m,r} exp\left(\sum_{i=1}^{n} b_i\, x_i\right).$$

$$\sum_{i=1}^{n} b_i = b + b_m + b_r + b_c + b_s + b_{mr} + b_{mc} + b_{ms} + b_{rc} + b_{rs} + b_{mrc} + b_{mrs} + b_{rcs}$$

where $I_{m,r,c,s}$ is the number of injuries observed in Stats19 from 2005-2015 in England for travel mode *m*, on road type *r*, with casualty mode *c*, and severity *s*. The corresponding expected number of injuries for this category is $\lambda_{m,r,c,s}$, which is a function of the corresponding distance D travelled by the road user m on road type r, and the expected number of injuries per km for the category *m,r,c,s*, which is a log-linear function of the coefficients b_i. These coefficients include an intercept term representing the expected number of injuries where all predictors take their baseline value, main effects b_m, b_r, b_c, b_s of each mode m, road type *r*, casualty mode *c* and severity *s*, all two-way interactions between these variables, and all three-way interactions, excluding the interaction between mode, casualty mode and severity. This was the most complex model that was computationally feasible to fit. The coefficients b_i are learnt via the glm function in R.

A second, similar model is fitted which also includes gender *g* as a predictor. Again, this includes all main effects, two-way and three-way interactions excluding the interaction between mode, casualty model and severity.

Confidence intervals are obtained by simulating a large sample of values from the multivariate normal distribution defined by the estimates and covariance matrix of the bi, transforming to a sample of values for the expected number of deaths per km for the group of interest, and determining the 90% quantiles of the sample.

2. Distance attribution

We attribute travel to road type by sequentially allocating a trip's distance first to minor urban (rural) roads, then urban (rural) major roads, then minor rural (urban) roads, then rural (urban) major roads, and the remainder to motorways, for an urban (rural) person. We learn the distance thresholds using a simple optimisation function to minimise the divergence between the RTS estimates and the NTS estimates scaled up to the whole population using ONS population estimates.

Any distance in excess of what is allocated to the first four road types is attributed to motorway, which is NA for bikes: any distance beyond the first three road types goes to the fourth road type.

Table 1: Apportionment of distance for different modes

		Car	Motorcycle	Van	Bike
1	Taken on rural minor road by rural resident	8.21932511	9.15403824	11.24042916	18.0172
2	Taken on rural major road by rural resident (after 1)	33.7572116	35.822129	37.19173417	10.0173
3	Taken on urban minor road by rural resident (after 2)	1.56744496	5.90344252	1.164378324	8.00045
4	Taken on urban major road by rural resident (after 3)	1.80821506	7.11639474	2.142441811	Remaining
5	Taken on motorway by rural resident (after 4)	Remaining	Remaining	Remaining	None
6	Taken on urban minor road by London resident	5.74588163	10.1791246	7.296514324	6.99975
7	Taken on urban major road by London resident (after 6)	24.2647224	24.3081001	23.8185963	15.0001
8	Taken on urban minor road by urban resident	5.20628762	10.3764801	7.918806022	4.6771
9	Taken on urban major road by urban resident (after 8)	8.39420246	13.6636638	10.71601837	4.54894
10	Taken on rural minor road by urban resident (after 7/9)	2.16186477	7.76158421	3.754233787	14.9999
11	Taken on rural major road by urban resident (after 10)	24.6250738	35.8507295	31.43879387	Remaining
12	Taken on motorway by urban resident (after 11)	Remaining	Remaining	Remaining	None

3. Supplementary results

Headline results with multi-vehicle collisions excluded:

Table 2: Total ORU fatalities by road-user type as a percentage of all fatalities on the road type

	Rural A	Urban A	Rural Minor	Urban Minor	Total
Car/Taxi	2731 (66.2%)	1646 (62.7%)	1549 (74.3%)	1658 (70.3%)	7584 (67.7%)
Van	308 (7.5%)	176 (6.7%)	206 (9.9%)	214 (9.1%)	904 (8.1%)
Lorry	900 (21.8%)	432 (16.5%)	202 (9.7%)	219 (9.3%)	1753 (15.7%)
Motorcycle	68 (1.6%)	97 (3.7%)	47 (2.3%)	57 (2.4%)	269 (2.4%)
Bus	115 (2.8%)	261 (9.9%)	69 (3.3%)	193 (8.2%)	638 (5.7%)
Cycle	6 (0.1%)	14 (0.5%)	12 (0.6%)	18 (0.8%)	50 (0.4%)
Total	4128	2626	2085	2359	11198

Note that were we to exclude multi-vehicle collisions (as in other literature) while the overall picture stays the same, motorised modes but not cycles look proportionally safer.

Figure 1: comparison of ORU fatality rates if multi-party collisions were excluded, with our main findings
