# Supplementary figures and images for: How does mode of travel affect risks posed to other road users? An analysis of English road fatality data, incorporating gender and road type
Source: Inj Prev. 2020 Apr 6;27(1):71–6. doi: 10.1136/injuryprev-2019-043534 (PMC7848050; doi:10.1136/injuryprev-2019-043534)

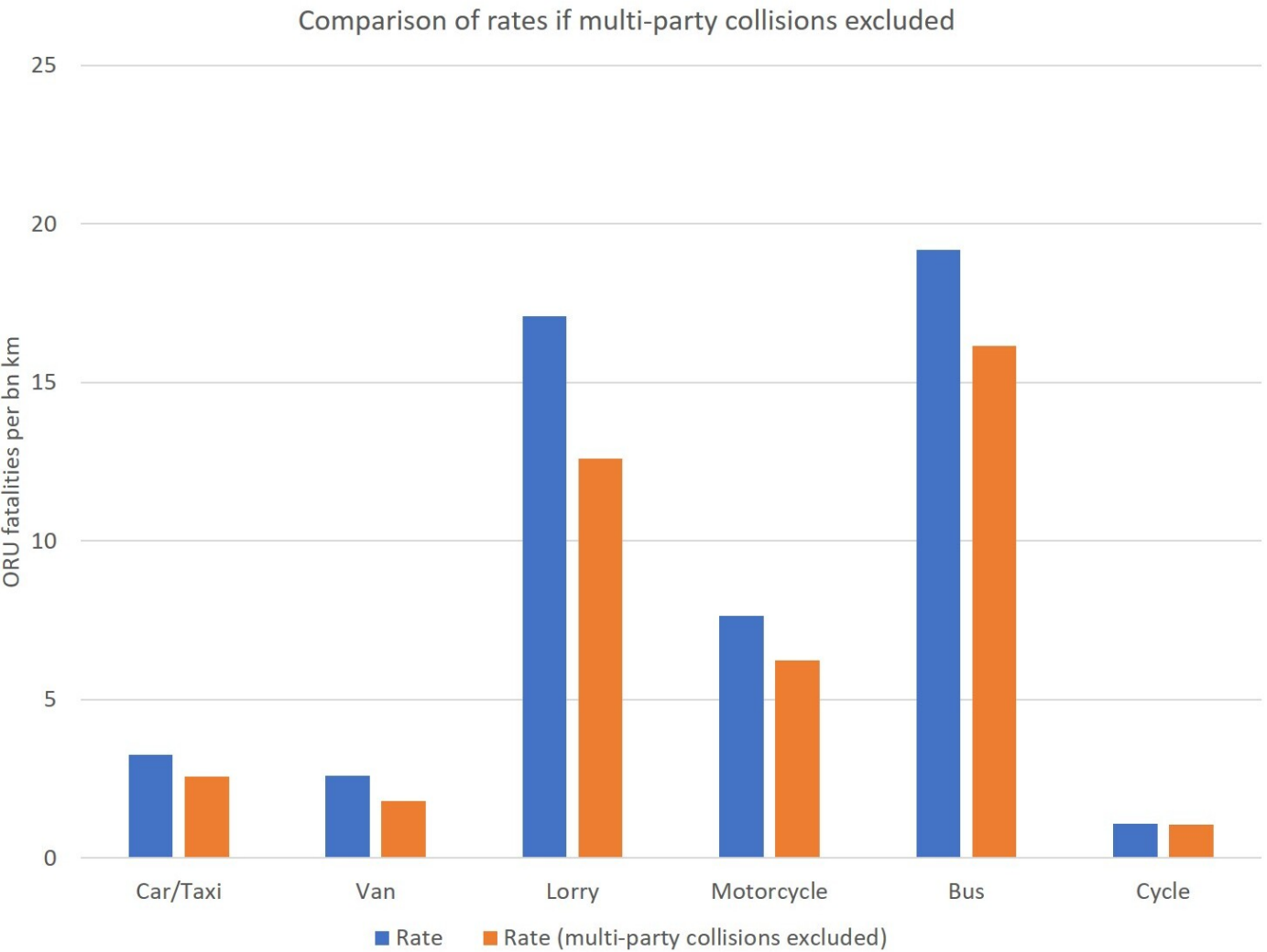

Supplement: Supplementary data [file injuryprev-2019-043534supp002.pdf]
